# Methacholine-Induced Cough in the Absence of Asthma: Insights From Impulse Oscillometry

**DOI:** 10.3389/fphys.2020.554679

**Published:** 2020-10-06

**Authors:** Nilita Sood, Nastasia V. Wasilewski, Andrew G. Day, Taylar Wall, Thomas Fisher, John T. Fisher, M. Diane Lougheed

**Affiliations:** ^1^Department of Medicine, School of Medicine, Faculty of Health Sciences, Queen’s University, Kingston, ON, Canada; ^2^Department of Biomedical and Molecular Sciences, School of Medicine, Faculty of Health Sciences, Queen’s University, Kingston, ON, Canada; ^3^Kingston General Health Research Institute, Kingston Health Sciences Centre, Queen’s University, Kingston, ON, Canada

**Keywords:** asthma, cough, cough variant asthma, chronic cough, deep inspiration, impulse oscillometry, methacholine, methacholine challenge test

## Abstract

**Introduction:**

The pathophysiologic differences between methacholine-induced cough but normal airway sensitivity (COUGH) and healthy individuals (CONTROL) are incompletely understood and may be due to differences in the bronchodilating effect of deep inspirations (DIs). The purpose of this study is to compare the bronchodilating effect of DIs in individuals with classic asthma (CA), cough variant asthma (CVA), and COUGH with CONTROL and to assess impulse oscillometry (IOS) measures as predictors of the bronchodilating effect of DIs.

**Methods:**

A total of 43 adults [18 female; 44.8 ± 12.3 years (mean ± SD); *n* = 11 CA, *n* = 10 CVA, *n* = 7 COUGH, *n* = 15 CONTROL] underwent modified high-dose methacholine challenge, with IOS and partial/maximal expiratory flow volume (PEFV/MEFV) maneuvers (used to calculate DI Index) to a maximum change (Δ) in FEV_1_ of 50% from baseline (MAX). Cough count and dyspnea were measured at each dose. The relation between IOS parameters and DI Index was assessed at baseline and MAX using multivariable linear regression analysis.

**Results:**

Cough frequency, dyspnea intensity, and baseline peripheral resistance (R5–R20) were significantly greater in COUGH compared with CONTROL (*p* = 0.006, *p* = 0.029, and *p* = 0.035, respectively). At MAX, the DI Index was significantly lower in COUGH (0.01 ± 0.36) compared with CA (0.67 ± 0.97, *p* = 0.008), CVA (0.51 ± 0.73, *p* = 0.012), and CONTROL (0.68 ± 0.45, *p* = 0.005). Fres and R5–R20 were independent IOS predictors of the DI Index.

**Conclusion:**

The bronchodilating effect is impaired in COUGH and preserved in mild CA, CVA, and CONTROL. Increased peripheral airway resistance and decreased resonant frequency are associated with a decreased DI Index. COUGH is a clinical phenotype distinct from healthy normals and asthma.

## Introduction

Asthma is a chronic inflammatory disorder of the airways associated with a variable degree of airway hyperresponsiveness and airflow obstruction, producing symptoms of chest tightness, wheeze, cough, and dyspnea. When cough presents as the sole or predominant symptom of asthma, it is described as cough variant asthma (CVA) (Glauser, 1972; Corrao et al., 1979). CVA is typically diagnosed when individuals with chronic cough have evidence of asthma on pulmonary function tests (reversible airflow obstruction or airway hyperresponsiveness to non-specific stimuli), and report resolution of their cough with standard asthma therapy (Irwin et al., 2006). However, the underlying pathophysiologic mechanisms differentiating classic asthma (CA) and CVA are not fully understood.

Recent studies have identified a group of individuals with chronic cough (and suspected CVA) who cough during high-dose methacholine challenge but have normal airway sensitivity (COUGH) (Wasilewski et al., 2015, 2018; Sood et al., 2018). During methacholine challenge testing, these individuals cough more frequently and develop significant dynamic hyperinflation, compared to individuals with CA (Wasilewski et al., 2015), and develop significant increases in esophageal pressures before a cough, which partially resolves following a deep inspiration (DI) and cough (Sood et al., 2018). This partial normalization is likely due to a combination of the bronchoprotective and bronchodilating effects of a DI (Ohkura et al., 2009), where an inhalation to total lung capacity (TLC) dilates the airways to minimize airflow limitation and subsequently protects against bronchoconstriction.

Since both maximal inspiratory and maximal forced expiratory maneuvers involve DIs, plethysmography and spirometry measurements reflect the combination of the airway smooth muscle response to the inhaled methacholine and the response to a DI. Impulse oscillometry (IOS), a variant of the forced oscillation technique, uses rectangular pressure waves superimposed on an individual’s tidal breathing to assess the degree of obstruction in the central and peripheral airways (LaPrad and Lutchen, 2008). Several studies have shown IOS to be more sensitive than spirometry for detecting peripheral airway abnormalities and assessing bronchodilator responses in asthma (Marotta et al., 2003; Kaminsky, 2011). Because IOS does not require DIs to generate data about the mechanical properties of the respiratory system, it assesses peripheral airway function in the absence of DIs (Navajas and Farré, 2001; Chapman et al., 2009) making it particularly valuable as a tool to tease out the significance of the bronchoprotective and bronchodilating effects of a DI.

Wasilewski et al. (2015) reported that the bronchodilating effect of DIs is preserved in individuals with CVA and CA. However, in the absence of comparison with healthy individuals, the clinical significance of the responses in the COUGH group remained uncertain. In order to tease out the clinical relevance of COUGH, we compared the physiologic responses and the bronchodilating effect of a DI in response to high-dose methacholine in CA, CVA, and COUGH to healthy individuals (without asthma, chronic cough, or asymptomatic airway hyperresponsiveness, CONTROL). We chose high-dose methacholine provocation to induce physiologic changes, especially for the COUGH and CONTROL groups where responses are often mild, even at the methacholine doses as high as 256 mg/mL. We hypothesized that the bronchodilating effect of a DI would be absent or impaired in individuals with CA, impaired in individuals with CVA and COUGH, and preserved in CONTROL. Another objective of this study was to investigate IOS measures of pulmonary resistance and reactance as potential determinants of DI-induced bronchodilation in our study population. Some preliminary results have been previously reported in the form of an abstract (Sood et al., 2017).

## Materials and Methods

### Participants

Participants with CA, CVA, and COUGH were recruited from patients (age 18–65 years) referred to a tertiary care cough clinic in Kingston, as previously described (Wasilewski et al., 2015, 2018). They were invited to participate if they had chronic cough because of suspected or proven CA or CVA. Healthy participants, aged 18–65 years, with no history of asthma, allergies (seasonal or otherwise), rhinitis/sinusitis, eczema, and/or chronic cough were recruited using print advertisements in Kingston, as previously described (Sood et al., 2018).

The study was approved by the Queen’s University Health Sciences Research Ethics Board and received a Letter of No Objection from Health Canada Therapeutic Products Directorate. The clinical trial was registered on www.clinicaltrials.gov (NCT01659476).

### Study Design

Participants had previously participated in other studies involving high-dose methacholine testing and had already been classified as CA, CVA, COUGH (Wasilewski et al., 2015, 2018), and CONTROL (Sood et al., 2018). The following definitions were used:

(a) CA: Episodic respiratory symptoms occurring in association with variable airflow obstruction and/or methacholine PC_20_ ≤ 16 mg/mL (Canadian Thoracic Society Asthma Guidelines; Lougheed et al., 2012);
(b) CVA: Asthma (PC_20_ ≤ 16 mg/mL) with chronic cough as the sole or predominant symptom, and history of response to asthma treatment (such as inhaled corticosteroids or 1 week trial of bronchodilator therapy; Irwin et al., 2006);
(c) COUGH: Chronic cough (Irwin et al., 2006) as the sole or predominant symptom and a negative methacholine challenge (PC_20_ > 16 mg/mL) with the presence of cough (Turcotte and Lougheed, 2011; Wasilewski et al., 2015); and
(d) CONTROL: No history of asthma, allergies (seasonal or otherwise), rhinitis/sinusitis, eczema and/or chronic cough, FeNO < 25 ppb, and a negative MCh challenge (PC_20_ > 16 mg/mL) (Sood et al., 2018).

After written informed consent, a detailed medical history was taken. All participants were screened for exclusion criteria, as previously described (Wasilewski et al., 2015, 2018; Sood et al., 2018). CONTROL participants completed the self-administered comorbidity questionnaire (Sangha et al., 2003) to assess any existing comorbidities. They had their exhaled fraction of nitric oxide (FeNO) measured to exclude baseline airway inflammation. Participants with no asthma or cough but with FeNO > 25 ppb were also excluded from the CONTROL group. Participants with CA, CVA, and COUGH completed the Mini Asthma Quality of Life Questionnaire (Juniper et al., 1999) and the Leicester Cough Questionnaire (Birring et al., 2003). Data for these questionnaires have been previously published (Wasilewski et al., 2015).

**FIGURE 1:**
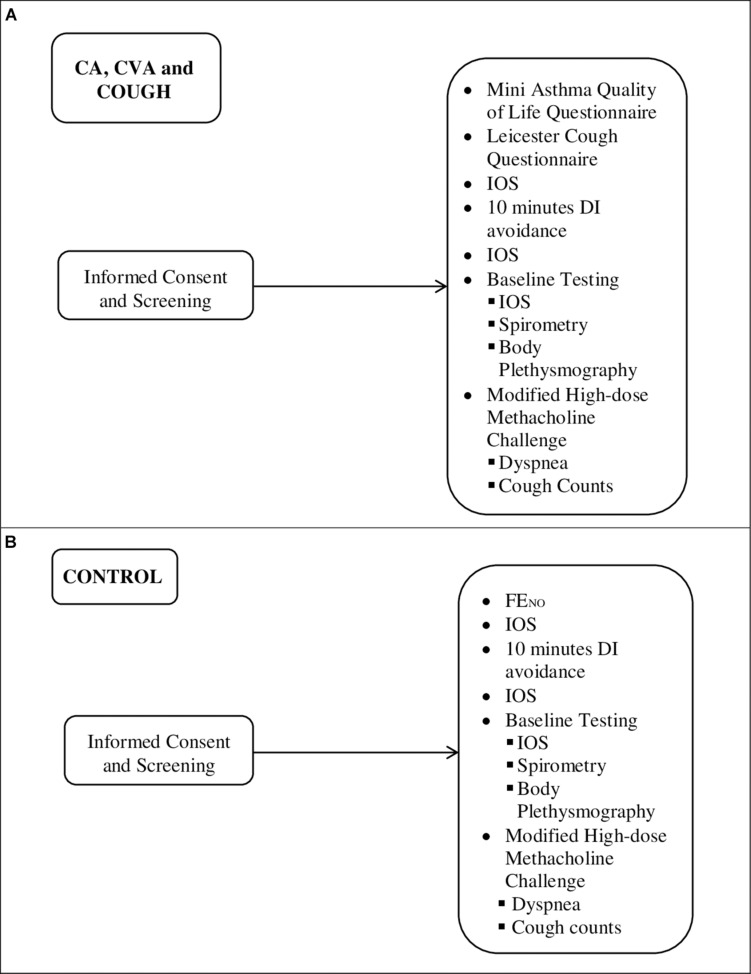
Outline of sequence of testing for **(A)** CA (*n* = 11), CVA (*n* = 10), and COUGH (*n* = 7) and **(B)** CONTROL (*n* = 15). FE_*NO*_, exhaled fraction of nitric oxide.

Participants then completed baseline pulmonary function testing, including IOS measurements, spirometry, body plethysmography, maximal and partial expiratory flow volume curves (MEFV and PEFV, respectively), followed by a modified high-dose methacholine challenge protocol (Figure 1). Short-acting bronchodilators were withheld for 8 h, long-acting bronchodilators beta-agonists were held 48 h, tiotropium bromide was withheld for 72 h, and leukotriene receptor antagonists for 96 h prior to testing as per the American Thoracic Society guidelines (Crapo et al., 2000). We did not collect information about use of antihistamines usage in the participants.

### Measurements

#### Exhaled Nitric Oxide Measurement

Only CONTROL participants had FeNO measured according to American Thoracic Society standards (Dweik et al., 2011), using NIOX MINO^®^ (Aerocrine AB, Solna, Sweden). FeNO measurements were performed prior to the high-dose methacholine test. After inhaling room air, participants exhaled from TLC (without nose clips) into the NIOX MINO^®^ device.

#### Pulmonary Function Testing

Spirometry, body plethysmography, and specific airway resistance were performed using 6200 Autobox DL (SensorMedics, Yorba Linda, CA, United States) according to the recommended techniques (Miller et al., 2005). Participants were trained in performing the lung volume maneuvers by a Registered Respiratory Therapist during the baseline testing. Lung volumes and specific airway resistance (sRaw) were determined by constant-volume body plethysmography. Panting frequency was standardized at 1 Hz to minimize the potential for frequency-dependent overestimation of thoracic gas volume during bronchoconstriction (Rodenstein and Stânescu, 1983). Inspiratory capacity (IC) was measured using body plethysmography. TLC was calculated as the sum of functional residual capacity (FRC) and IC. Dynamic hyperinflation was assessed using change in residual volume (RV) and RV/TLC.

The predicted values used for spirometry, lung volumes, and airway resistance were by Briscoe and Dubois (1958), Goldman and Becklake (1959), and Morris et al. (1971), respectively.

#### Methacholine Challenge

High-dose methacholine challenge testing was performed using Provocholine^®^ (methacholine chloride; Methapharm Ltd., Brantford, ON, Canada) according to a standardized high-dose tidal breathing protocol (Sterk et al., 1985). Isotonic saline (0.9%) was first administered as a control, followed by participants inhaling methacholine doses for 2 min while seated upright. The methacholine doses were administered within approximately 5 min intervals of each other (Crapo et al., 2000). Immediately following each methacholine dose, IOS measurements were obtained. Subsequently, PEFV and MEFV maneuvers were performed according to a published method (Sterk et al., 1985) to assess the bronchodilating effect of DIs (Supplementary Figure S1). Plethysmographic measurements were obtained at baseline, after inhalation of nebulized normal saline (0.9%), and at the maximum response to methacholine (MAX). MAX was defined as any of the following: (i) a decline FEV_1_ to 50% of the baseline value; (ii) the mean value of FEV_1_ on a response “plateau” (defined as a change in FEV_1_ of <5% over two or more dose steps after a fall of >10% from the baseline value); (iii) the change in FEV_1_ after the highest methacholine dose (256 mg/mL) had been delivered; or (iv) the change in FEV_1_ at the cessation of testing at the participant’s request.

After testing, bronchoconstriction, if present, was reversed with inhaled salbutamol (200 μg every 10 min by metered dose inhaler and spacer), until the FEV_1_ was within 10% of the baseline value. The provocative concentration of methacholine causing a 20% fall in FEV_1_ from baseline (PC_20_) was interpolated from log_10_ dose–response curves.

#### Symptom Evaluation

Cough was defined as an audible expiratory maneuver against a closed glottis (Morice, 2008; Chung et al., 2009). Coughs during the high-dose methacholine test were manually counted and recorded using a microphone. Dyspnea was defined as previously described (Lougheed et al., 1993). Participants were asked to rate their difficulty breathing in, breathing out, and their overall difficulty breathing using the modified Borg scale (Borg, 1982).

Cough frequency and dyspnea were assessed at baseline, after isotonic saline, and at each methacholine dose step. Any coughs occurring in response to methacholine (not including any coughs at baseline, after isotonic saline, and after salbutamol inhalation) were summed for analysis.

#### Impulse Oscillometry

Impulse oscillometry measurements were performed prior to spirometry, immediately after the isotonic saline/methacholine dose (Supplementary Figure S1). Measurements were obtained using the Jaeger Masterscreen IOS system (Erich Jaeger, Hoechberg, Germany), as previously described (Oostveen et al., 2003; Borrill et al., 2005). During each acquisition, participants were instructed to breathe through a mouthpiece in a relaxed manner while seated upright, wearing a nose clip, and supporting their cheeks using both hands. Participants were trained by a Registered Respiratory Therapist not to occlude the mouthpiece with their tongue and monitored during data acquisition. Each participant took about 60 s for the IOS measurement. Due to time constraints, IOS measurements were performed twice at each dose step. Coherence values used were >0.6 at 5 Hz and ≥0.8 at 10 Hz or more (Brashier and Salvi, 2015). After each measurement, tidal volume, respiratory system resistance (Rrs), and reactance (Xrs) were displayed on the computer screen. All data were visually inspected and checked for artifacts, such as irregular breathing, hyperventilation, leakages, or swallowing. Measurements with artifacts were discarded and, if possible, repeated.

#### Bronchodilating Effect

The bronchodilating effect of DIs was examined by comparing the flow difference between the PEFV and MEFV at an isovolume of 40% of the control vital capacity (PEF_40_ and MEF_40_, respectively) (Ohkura et al., 2010). Specifically, these were used to calculate DI index (Fujimura et al., 1990; Ohkura et al., 2010), as follows:

(MEF-40PEF)40/PEF40

### Statistical Analysis

All data are expressed as mean ± SD unless indicated otherwise. The analyses of responses to high-dose methacholine challenge were performed using SPSS version 22.0.0 (IBM Corporation, Chicago, IL, United States). Previously published data (Wasilewski et al., 2015) were used to compare the responses to high-dose methacholine for CONTROL participants (*n* = 15) to individuals CA (*n* = 11), CVA (*n* = 10), and COUGH (*n* = 7). For this comparison, the raw values for IOS parameters were used to assess between-group differences. Between-group comparisons were made using ANOVA with Bonferroni correction for multiple comparisons or Kruskal–Wallis tests with *post hoc* Mann–Whitney *U* tests. Within-group comparisons were made using paired *t*-tests or Wilcoxon signed-rank tests (paired).

#### Assessing IOS Measures as Predictors of DI Index

The goal of this correlation and regression analysis was to assess if IOS measures of pulmonary resistance and reactance predict DI index independently of age, sex, height, BMI, spirometry, and lung volume. The correlation and linear regression analyses were performed using SAS version 9.4 (SAS Institute Inc., Cary, NC, United States). Only measures at baseline and at MAX were used. Detailed description of the analysis is presented in the Supplementary Material.

## Results

Tables 1, 2 contain the baseline characteristics for the CA, CVA, COUGH, and CONTROL groups.

**TABLE 1:** Participant characteristics and baseline lung function measures.

	**CA**	**CVA**	**Cough**	**Control**	***P*-value**
	**(*n* = 11)**	**(*n* = 10)**	**(*n* = 7)**	**(*n* = 15)**	
Age (years)	39.811.9^§^	53.09.9	40.911.7^§^	31.47.2^§^	**0.004**
Sex (% female)	72.7	80.0	71.4	60.0	0.766
BMI (kg/m^2^)	32.86.3	28.27.0	26.77.5	26.27.4	0.120
Smoking history (pack year)	5.03.5^(*n* = 2)^	1.2^(n=1)^	5.76.1^(*n* = 2)^	5.0^(n=1)^	N/A
Plateau (%)	45.5	40.0	71.4	40.0	0.556
Allergies (%)	63.6	72.7	42.9	0.0^ψ§^	**0.001**
Eczema (%)	9.0	20.0	14.3	0.0	0.387
Rhinitus/sinusitis (%)	45.5	36.4	57.1	0.0^#^	**0.016**
PC_20_ MCh (mg/mL)	2.192.32	5.594.09	49.317.0^(*n* = 4)#^	93.485.4^(*n* = 4)#^	< **0.001**
MAX MCh (mg/mL)	7.365.97	50.045.7	137.186.1^ψ§^	256.0^ψ§^	< **0.001**
**Medication usage**					
SABA use (%)	90.1	70.0	71.4	0.0^ψ§^^#^	< **0.001**
ICS use (%)	72.7	40.0	42.9	0.0^ψ§^^#^	< **0.001**
Combination ICS/LABA (%)	45.5	20.0	28.6	0.0^ψ^	**0.045**
**Sensory responses**					
Borg overall	0.10.2^(*n* = 10)^	0.50.7^(*n* = 8)^	0.81.1^(*n* = 6)^	0.0	**0.034**
**Spirometry**					
FEV_1_ (%pr)	88.219.1	82.712.2	99.419.8	106.910.9^ψ§^	**0.002**
FVC (%pr)	99.919.8	95.815.4	104.720.4	103.422.7	0.768
FEV_1_/FVC (%pr)	89.96.2	92.29.0	96.49.5	98.48.2	0.233
FEF_25__–__75%_ (%pr)	70.023.1	74.719.9	90.436.9	93.623.5	0.081
**Lung volumes**					
TLC (%pr)	102.619.4	100.817.1	105.77.26	103.113.8	0.935
RV (%pr)	107.345.8	109.824.8	116.122.4	92.942.1	0.504
IC (%pr)	125.436.5	115.922.9	104.512.3	108.016.2	0.227
**Impulse oscillometry**					
R5 (cmH_2_O/L/s)	5.441.56	4.341.21	4.071.95	3.601.00^ψ^	**0.025**
R20 (cmH_2_O/L/s)	4.161.34	3.480.98	3.361.39	3.520.93	0.424
R5–R20 (cmH_2_O/L/s)	1.280.52	0.860.39	0.740.70	0.260.14^ψ§^	< **0.001**
X5 (cmH_2_O/L/s)	−1.900.86	−1.340.26	−1.361.26	−0.840.47^ψ^	**0.003**
AX (cmH_2_O/L)	10.436.39	5.352.86	5.549.75	1.621.46^ψ^	**0.001**
Fres (Hz)	17.173.27	14.172.10	11.316.19^ψ^	8.671.73^ψ§^	< **0.001**
**DI Index**	−0.010.27	−0.200.27	−0.290.16	−0.140.15	0.054
**LCQ scores**					
Physical domain	6.50.5	5.11.0^ψ^	5.31.1	−	0.031*
Psychological domain	6.80.1	4.61.4^ψ^	5.41.1	−	0.004*
Social domain	6.70.2	4.61.2^ψ^	4.91.3^ψ^	−	0.002*
Total score	19.90.9	14.43.4^ψ^	15.63.4^ψ^	−	0.002*
**Mini-AQLQ scores**					
Symptoms	5.90.8	5.11.0	5.71.4	−	0.305*
Activity limitation	6.60.4	6.21.1	5.81.5	−	0.347*
Emotional limitation	6.30.8	5.91.2	5.50.9	−	0.422*
Environmental limitation	6.20.7	5.01.2	4.81.4	−	0.059*
Overall score	6.20.5	5.51.2	5.51.0	−	0.181*

All values are mean ± SD, except ^#^ denoting median ± median absolute deviation and % denoting the proportion of participants. Bolded values indicate significant differences. BMI, body mass index; CA, classic asthma; COUGH, methacholine-induced cough but normal airway sensitivity; CVA, cough variant asthma; CONTROL, healthy participants; MCh, methacholine; PC_20_, provocative concentration eliciting a 20% decline in FEV_1_ from baseline; MAX MCh, methacholine dose eliciting maximum decline in FEV_1_ from baseline; ICS, inhaled corticosteroid; LABA, long-acting ß2-agonist; SABA, short-acting ß2-agonist; FEV_1_, forced expiratory volume in 1 s; FVC, forced vital capacity; FEV_1_/FVC, forced expiratory volume in 1 s divided by forced vital capacity; FEF_25__–__*75*%_, mean forced expiratory flow during the middle half of the forced vital capacity; IC, inspiratory capacity; LCQ, Leicester Cough Questionnaire; Mini-AQLQ, Mini-Asthma Quality of Life Questionnaire; RV, residual volume; TLC, total lung capacity; R5, resistance at 5 Hz (total respiratory resistance); R5–R20, frequency dependence of Rrs (R5 minus R20, peripheral respiratory resistance); R20, resistance at 20 Hz (central respiratory resistance); X5, reactance at 5 Hz (peripheral reactance); AX = area under the reactance curve below resonant frequency; Fres, resonant frequency; DI, deep inspiration. ^ψ^*p* < 0.05 compared with CA. ^§^*p* < 0.05 compared with CVA. *p-values were calculated using ANOVA with *post hoc* Bonferroni comparisons for CA, CVA, and COUGH only.

**TABLE 2:** Log-transformed impulse oscillometry measures.

	**CA**	**CVA**	**Cough**	**Control**	***P*-value**
	**(*n* = 11)**	**(*n* = 10)**	**(*n* = 7)**	**(*n* = 15)**	
**Baseline**					
Log (R5)	1.650.31	1.430.31	1.320.44	1.260.27^ψ^	**0.027**
Log (R20)	1.380.33	1.210.31	1.140.39	1.240.26	0.427
Log (R5–R20)	0.160.45	−0.230.41	−0.550.71	−1.530.72^ψ§#^	< **0.001**
−Log (−X5)	−0.540.49	−0.270.20	−0.090.64	0.420.57^ψ§^	< **0.001**
Log (AX)	2.120.76	1.580.43	0.911.16^ψ^	−0.071.01^ψ§^	< **0.001**
Log (Fres)	2.820.20	2.640.13	2.330.46^ψ^	2.130.23^ψ§^	< **0.001**
**Max**					
Log (R5)	2.060.28^‡‡^	1.850.33^‡‡^	1.770.49^‡‡^	1.790.26^‡‡^	0.172
Log (R20)	1.340.24	1.220.20	1.360.34	1.490.25^‡‡^	0.099
Log (R5–R20)	1.360.41^‡‡^	1.040.52^‡‡^	0.381.03^ψ^	0.340.78^ψ^	**0.001**
−Log (−X5)	−1.680.60^‡‡^	−1.450.52^‡‡^	-0.620.93ψ⁢§(n=6)‡‡	-0.620.43ψ⁢§‡‡	< **0.001**
Log (AX)	3.730.73^‡‡^	3.480.73^‡‡^	2.241.38ψ⁢§‡‡	2.340.83ψ⁢§‡‡	< **0.001**
Log (Fres)	3.300.28^‡‡^	3.220.20^‡‡^	2.840.43ψ‡‡	2.920.31ψ‡‡	**0.003**

*All values are mean ± SD. Bolded values indicate significant differences. For definitions of abbreviations, see Table 1. ^ψ^*p* < 0.05 compared with CA. ^§^*p* < 0.05 compared with CVA. ^#^*p* < 0.05 compared with COUGH. ^‡‡^*p* < 0.05 compared with baseline using paired *t*-test.*

### Baseline Pulmonary Function

At baseline, the %predicted FEV_1_ for COUGH was comparable to both CA and CVA, and significantly lower than CONTROL. There were no other significant differences in the baseline spirometry and lung volume measures within groups. Baseline FeNO for CONTROL participants was <25 ppb (16.3 ± 5.3 ppb).

At baseline, R5–R20 was comparable between the CA, CVA, and COUGH groups, and significantly elevated compared to CONTROL (Supplementary Table S1). All other baseline IOS measures were comparable between COUGH and CONTROL, and COUGH and CVA. However, COUGH had significantly lower AX and Fres compared to CA (Table 1 and Supplementary Table S1). Participants with CA and CVA also had elevated AX and Fres compared to CONTROL at baseline.

### Responses to High-Dose Methacholine

Group responses to high-dose methacholine are presented in Tables 2–4. Cough frequency in COUGH (32.0 ± 27.1) was comparable to CVA (33.0 ± 27.1; *p* = 0.775) and significantly higher than both CA (1.7 ± 2.9; *p* = 0.004 and 0.001 for COUGH and CVA, respectively) and CONTROL (5.6 ± 3.7; *p* = 0.006 and 0.001 for COUGH and CVA, respectively). Similarly, overall dyspnea intensity in COUGH (2.8 ± 1.8) was significantly higher than CONTROL (0.5 ± 0.9; *p* = 0.029), and comparable to that experienced by CA (2.7 ± 1.7; *p* = 0.631) and CVA (2.1 ± 1.2; *p* = 0.728). For participants with CA, CVA, and COUGH, dyspnea intensity on the Modified Borg Scale corresponded to “Moderate” at MAX.

**TABLE 3:** Select responses during the modified high-dose methacholine challenge.

	**CA**	**CVA**	**Cough**	**Control**	***P*-value**
	**(*n* = 11)**	**(*n* = 10)**	**(*n* = 7)**	**(*n* = 15)**	
**Sensory responses**					
Cough frequency	1.72.9^(*n* = 9)^	33.024.0‡‡(n=9)	32.027.1^‡‡^	5.63.7	< **0.001**
Borg overall	2.71.7^(*n* = 10)^	2.12.2^(*n* = 8)^	2.81.8^(*n* = 6)^	0.50.9	**0.004**
**Spirometry**					
%ΔFEV_1_	−37.09.5	−34.19.2	−21.615.1	−14.610.5^‡‡^	< **0.001**
ΔFEV_1_ (%pr)	−33.313.9^‡‡^	−28.49.0^‡‡^	−20.914.1^‡‡^	−15.812.2^‡‡^	**0.006**
ΔFVC (%pr)	−27.99.6	−21.88.9	−12.214.4	−6.34.3	< **0.001**
ΔFEV_1_/FVC (%pr)	−12.36.2^‡‡^	−15.03.9^‡‡^	−10.610.3^‡‡^	−6.43.5	**0.005**
ΔFEF_25__–__75%_ (%pr)	−37.517.7^‡‡^	−41.623.4^‡‡^	−31.516.4^‡‡^	−28.215.5^‡‡^	0.303
**Lung volumes**					
ΔTLC (%pr)	0.92.2	−0.50.8	−0.50.6	0.36.7	0.854
ΔRV (%pr)	42.113.4^‡‡^	21.212.9^‡‡^	12.018.9	7.816.9	< **0.001**
ΔRV/TLC (%pr)	35.712.8^‡‡^	18.911.6^‡‡^	10.115.4	7.213.7	< **0.001**
ΔIC (%pr)	−35.736.8^‡‡^	−24.015.5^‡‡^	−12.98.4^‡‡^	−12.115.36	0.054
**Impulse oscillometry**					
ΔR5 (cmH_2_O/L/s)	2.671.20^‡‡^	2.331.70^‡‡^	2.482.03^‡‡^	2.541.60^‡‡^	0.971
ΔR20 (cmH_2_O/L/s)	−0.230.68	−0.020.90	0.770.81^‡‡^	1.010.81^‡‡^	< **0.001**
ΔR5–R20 (cmH_2_O/L/s)	2.891.41^‡‡^	2.341.59^‡‡^	1.702.10	1.351.06^‡‡^	< **0.001**
ΔX5 (cmH_2_O/L/s)	−4.302.71^‡‡^	−3.522.69^‡‡^	-1.522.35‡‡(n=6)	−1.300.88^‡‡^	< **0.001**
ΔAX (cmH_2_O/L)	40.5226.4^‡‡^	33.7826.8^‡‡^	16.3325.1	13.2813.97^‡‡^	**0.002**
ΔFres (Hz)	10.725.65^‡‡^	11.235.49^‡‡^	7.334.38^‡‡^	10.685.51^‡‡^	0.343

*All values are mean ± SD. Δ = change from baseline to MAX. Bolded values indicate significant differences. For definitions of abbreviations, see Table 1. ^‡‡^*p* < 0.05, compared to baseline using paired *t*-test or Wilcoxon signed-rank test, as appropriate.*

**TABLE 4:** ANOVA results of responses during the modified high-dose methacholine challenge.

	**CA vs. CVA**	**CA vs. Cough**	**CA vs. Control**	**CVA vs. Cough**	**CVA vs. Control**	**Cough vs. Control**	***P*-value**
**Sensory responses**							
Cough frequency	**0.001**	**0.004**			**0.001**	**0.006**	< **0.001**
Borg overall			**0.010**			**0.029**	**0.004**
**Spirometry**							
%ΔFEV_1_		**0.033**	< **0.001**		**0.001**		< **0.001**
ΔFEV_1_ (%pr)			**0.006**				**0.006**
ΔFVC (%pr)		**0.005**	< **0.001**		**0.001**		< **0.001**
ΔFEV_1_/FVC (%pr)					**0.005**		**0.005**
ΔFEF_25__–__75%_ (%pr)							0.303
**Lung volumes**							
ΔTLC (%pr)							0.854
ΔRV (%pr)	**0.023**	**0.002**	< **0.001**				< **0.001**
ΔRV/TLC (%pr)	**0.038**	**0.002**	< **0.001**				< **0.001**
ΔIC (%pr)			0.057				0.054
**Impulse oscillometry**							
ΔR5 (cmH_2_O/L/s)							0.971
ΔR20 (cmH_2_O/L/s)		**0.034**	**0.002**		**0.010**		< **0.001**
ΔR5–R20 (cmH_2_O/L/s)			**0.002**				< **0.001**
ΔX5 (cmH_2_O/L/s)		**0.008**	**0.001**	**0.033**	**0.003**		**< 0.001**
ΔAX (cmH_2_O/L)		**0.022**	**0.006**	**0.011**	**0.002**		**0.002**
ΔFres (Hz)							0.343

*Δ = change from baseline to MAX. Bonferroni corrected *p*-values located within columns represent the significance between groups. Blank spaces represent non-significant differences between groups. Bolded values indicate significant differences. For the definitions of abbreviations, see Table 1.*

The percent change in FEV_1_ (%Δ FEV_1_) was comparable in COUGH and CONTROL, but significantly lower compared to CA. However, the ΔFEV_1_ (L) was significantly greater in COUGH (−0.70 ± 0.50 L) compared to CONTROL (−0.58 ± 0.41 L, *p* = 0.042). All groups had significant changes in the mid-to-late flows (% predicted, %pr) from baseline, but these were comparable between groups (Table 2). Compared to baseline, participants with CA, CVA, and COUGH developed significant dynamic hyperinflation [IC (%pr)], but only CA and CVA developed significant gas trapping [RV (% pr) and RV/TLC (%pr)]. IC (%pr) decreased more in CA and CVA, compared with COUGH and CONTROL, although the difference between groups was only of borderline statistical significance (*p* = 0.054). Gas trapping was comparable in COUGH, CVA, and CONTROL, but significantly greater in CA.

Most IOS parameters increased significantly from baseline to MAX in our four groups. Like CONTROL, the change in total resistance (R5) in COUGH could be attributed to a significant change in central resistance (R20). At MAX, COUGH had comparable R5–R20 to CONTROL, which was significantly lower than that in CA. COUGH also had comparable Fres, X5, and AX measures at MAX compared with CONTROL, but significantly different from CA and CVA. ΔX5 and ΔAX were also similar in CONTROL and COUGH groups.

### Bronchodilating Index

**FIGURE 2:**
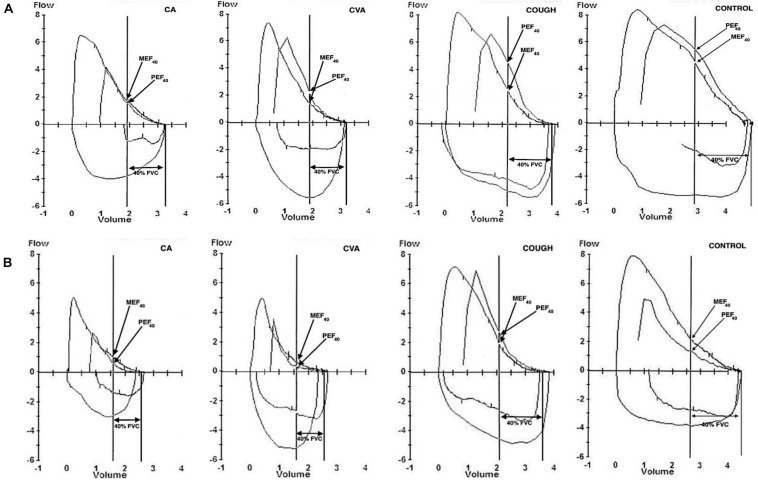
Representative partial and maximal expiratory flow-volume loops at **(A)** baseline and **(B)** MAX in CA, CVA, COUGH, and CONTROL. CA, classic asthma; CVA, cough variant asthma; COUGH, methacholine-induced cough but normal airway sensitivity; CONTROL, healthy normal participants; FVC, forced vital capacity; MEF_40_, expiratory flow at 40% of FVC from maximal curve; PEF_40_, expiratory flow at 40% of forced vital capacity from the partial curve.

Figure 2 shows representative PEFV/MEFV curves for CA, CVA, COUGH, and CONTROL at baseline and MAX, respectively, used to calculate the DI index. The DI index was comparable for all four groups at baseline (Table 1). The change in the DI index from baseline to MAX was significant in all four groups (*p* = 0.04, *p* = 0.01, *p* = 0.008, and *p* < 0.001 for CA, CVA, COUGH, and CONTROL, respectively).

**FIGURE 3:**
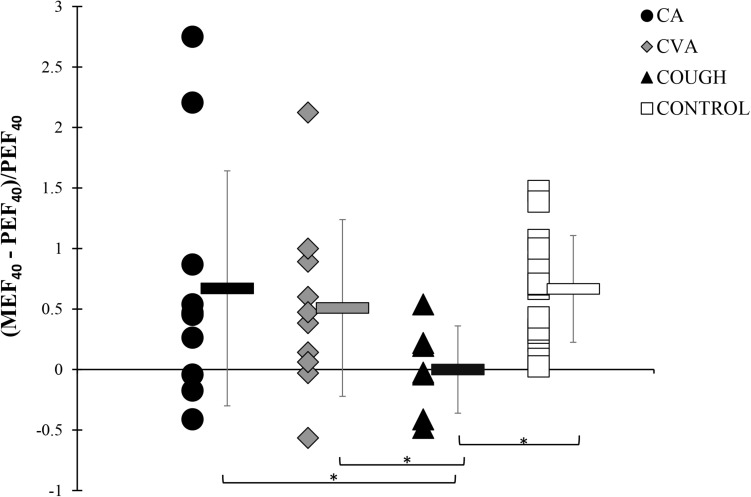
DI indices at MAX during the modified high-dose methacholine challenge in CA, CVA, COUGH, and CONTROL. * denotes *p* < 0.05. CA, classic asthma; COUGH, methacholine-induced cough but normal airway sensitivity; CONTROL, healthy normal participants; CVA, cough variant asthma; DI, deep inspiration; MEF_40_, expiratory flow at 40% of the forced vital capacity from the full forced vital capacity maneuver; MAX, maximal administered dose of methacholine; PEF_40_, expiratory flow at 40% of forced vital capacity from the partial curve.

At MAX, although positive, the DI index for COUGH group (0.01 ± 0.36) was significantly lower compared to the CA (0.67 ± 0.97), CVA (0.51 ± 0.73), and CONTROL (0.67 ± 0.44) groups, indicating that the bronchodilating effect in COUGH was impaired (Figure 3).

### Assessing IOS Measures as Predictors of DI Index

When assessing the IOS measures [log(R5), log(R20), log(R5–R20), −log(−X5), log(AX), log(Fres)] as predictors of DI-index, we attempted to adjust for age, sex, height, BMI, spirometry measures, and lung volume measures. The correlations for the variables are summarized in Supplementary Table S1. The first five principal components (PCs) of these 22 variables had eigenvalues ≥ 1.0 and collectively explained 85% of the variance in these 22 variables and 32% of the variance in the DI-index (Supplementary Table S2). For comparison, age, sex, height, and weight collectively explained 18% of the variance in the DI-index.

Table 5 provides the Pearson correlations between IOS measures and the DI index. After controlling for the five PCs described above, the partial correlations ranged from 0.28 for log(R20) up to 0.59 for log(Fres) (Table 5). All the partial correlations except R20 had a false discover rate < 5%. Although the estimated magnitudes of the unadjusted Pearson correlations were clinically meaningful, they were consistently smaller than the partial correlations and generally not statistically significant.

**TABLE 5:** Correlation between impulse oscillometry and DI (*n* = 42).

	**Correlation coefficient**	**Raw *P*-value**	**False discovery rate**
**Unadjusted pearson correlation coefficients**			
Log(R5)	0.24	0.115	0.173
LogR20)	0.26	0.102	0.173
Log(R5–R20)	0.20	0.188	0.226
Log(AX)	0.26	0.099	0.173
−Log(−X5)	–0.17	0.280	0.280
Log(Fres)	0.32	0.041	0.173
**Partial pearson correlations controlling for age, sex, height, and BMI**			
Log(R5)	0.32	0.050	0.066
Log(R20)	0.20	0.239	0.239
Log(R5–R20)	0.33	0.044	0.066
Log(AX)	0.34	0.041	0.066
−Log(−X5)	–0.32	0.055	0.066
Log(Fres)	0.44	0.007	0.042
Partial pearson correlations controlling for first five PCs of 22 covariates*			
Log(R5)	0.41	0.015	0.023
Log(R20)	0.28	0.101	0.101
Log(R5–R20)	0.41	0.015	0.023
Log(AX)	0.51	0.002	0.006
−Log(−X5)	–0.48	0.037	0.044
Log(Fres)	0.59	0.0002	0.001

PCs, principal components; R5, resistance at 5 Hz (total respiratory resistance); R5–R20, frequency dependence of Rrs (R5 minus R20, peripheral respiratory resistance); R20, resistance at 20 Hz (central respiratory resistance); X5, reactance at 5 Hz (peripheral reactance); AX, area under the reactance curve below resonant frequency; Fres, resonant frequency. *22 covariates include age, sex, height, BMI, 11 spirometry variables (FEV_1_, ΔFEV_1_, %prΔFEV_1_, FVC, ΔFVC, %prΔFVC, FEV_1_/FVC, PEF, FEF_*50*%_, FEF_25__–__*75*%_, FEF_*75*%_), and seven lung volume measures (TLC, RV, RV/TLC, FRC, ERV, IC, IC/TLC). False discovery rates were calculated within each adjustment group to account for six tests performed.

Without adjustment for other covariates, the six IOS variables explained 33% (adjusted *R*^2^ = 21%) of the variance in the DI index (*p* = 0.027) (see Supplementary Table S3 for details of the multiple regression models). After controlling for the five PCs, the selection method described in the Supplementary Methods retained log(R5–R20) and log(Fres) from the six IOS variables.

The stability of the model selection across 10,000 bootstrap samples is described in Supplementary Table S4. After controlling for the five PCs, log(Fres) was selected in 90% of the models and log(R5–R20) in 47%.

## Discussion

Our main finding was that the DI index for COUGH was significantly lower compared to CA, CVA, and CONTROL, suggesting that the bronchodilating effect of DIs is impaired in individuals with COUGH, but preserved in CA, CVA, and health. We have recently shown that individuals with COUGH develop significant small airway obstruction, but experience only a partial normalization of end-expiratory esophageal pressures and mid-to-late flows after a cough (Sood et al., 2018). This partial normalization could be attributed to impairment of the bronchodilating effect of DIs in COUGH. Overall, the methacholine-induced bronchoconstriction [ΔFEV_1_ (%pr)] is consistent with the concept of a continuum across for the four groups, from CA to CVA, to COUGH and CONTROL. Interestingly, the bronchodilating effect of DIs has recently been found to be reduced in children with exercise-induced (indirect) vs. methacholine-induced (direct) bronchoprovocation (Ioan et al., 2017). Ioan et al. (2017) suggest that bronchoconstriction and airway inflammation have “opposite” effects on the impact of DIs on bronchodilation. It would be of interest to tease out the bronchodilating ability of the COUGH group comparing direct vs. indirect bronchoprovocation tests.

COUGH participants developed dyspnea comparable to CA and CVA in response to high-dose methacholine, despite lower levels of bronchoconstriction and gas trapping. The magnitude of the breathlessness scores in our COUGH participants was slightly higher than what has been reported previously in CA (Lougheed et al., 2006), yet their declines in FEV_1_ and FVC were comparable to those in healthy participants. In the study by Lougheed et al. (2006), the IC for asthma participants decreased by 600 mL at PC_20_, whereas in our COUGH participants, IC decreased by 340 mL at the maximum methacholine dose. COUGH participants also developed cough comparable to CVA, significantly higher compared to both CA and CONTROL groups. Recently, Ohkura et al. (2012) showed that the cough response to bronchoconstriction was heightened in individuals with CVA, in the absence of increased cough reflex sensitivity to capsaicin. Despite being on controller medication, which is known to decrease cough in CVA (Ohkura et al., 2012), cough counts were higher in our CVA and COUGH groups. That participants with COUGH developed cough comparable to CVA and have an increased perception of dyspnea are important novel findings, which further support our hypothesis that COUGH is a clinically distinct phenotype, separate from CVA (based on PC_20_) and from health (based on cough frequency and dyspnea). Our observations also raise the question of whether lung or upper airway afferents contribute to the sensations in the COUGH group (Mazzone and Undem, 2016).

COUGH patients present a unique clinical challenge, both diagnostically and therapeutically. A negative methacholine test is often used to rule out current asthma as the cause of chronic cough (Brown et al., 2007). However, the ongoing respiratory symptoms, particularly chronic cough, are associated with significant morbidity and reduced quality of life (Ford et al., 2006). These patients are often prescribed inhaled rescue bronchodilators and/or asthma controller medications, while they undergo extensive testing to investigate for asthma-like conditions (such as non-asthmatic eosinophilic bronchitis). It is possible that individuals with COUGH lie on a continuum of airway response severity, with the normal healthy response on one end and the impaired response in airway disorders, such as CVA and CA on the other. The underlying pathophysiological mechanisms remain unknown, although the clinical phenotype and presence of some baseline peripheral airway abnormality may reflect the impact of early “subclinical” airway remodeling and could prove useful in early disease detection. Further research is needed to understand whether COUGH represents a distinct disease state or a “pre-pathological” phenotype at risk of deteriorating over time to develop CVA or severe CA.

### Assessing IOS Measures as Predictors of DI Index

To our knowledge, this is the first study that examines the relationship between IOS parameters and DI Index. Our results demonstrate log(Fres) and log(R5–R20) are the strongest IOS predictors for DI index, with an increase in R5–R20 or decrease in Fres, resulting in a significant decrease in DI Index. Recently, Foy et al. (2019) used CT-guided computational modeling to show that constriction of peripheral airways (diameter < 1.39 mm) plays a dominant role in increasing R5–R20 values. We found that participants with COUGH had elevated baseline R5–R20, comparable to CA and CVA, indicating some level of baseline peripheral airway abnormality. This is a novel observation in COUGH, given that this group does not meet the current diagnostic criteria for either CA or CVA.

Preliminary results from the ATLANTIS study have demonstrated peripheral airway resistance (R5–R20) and hyperinflation (RV/TLC) to be strongly positively correlated with the prevalence of small airway disease in asthma (Postma et al., 2019). This finding is replicated in our model with R5–R20 and RV/TLC as significant predictors of a decreased DI Index. We also found Fres to be a strong predictor of an increased DI Index. Fres is thought to mark the transition from small airway obstruction (capacitive dominance at low frequencies) to large airway obstruction (inertial dominance at high frequencies) (Brashier and Salvi, 2015). Individuals with CA and CVA had elevated Fres at baseline, which reflects underlying peripheral airway obstruction present in these groups. Interestingly, at baseline, individuals with COUGH had borderline elevated Fres values, indicating some level of baseline peripheral airway obstruction in this group. Future studies using IOS using direct and indirect airway bronchoprovocation would allow further understanding of the involvement of peripheral airways and the effects of DIs in COUGH.

### Limitations

The small sample size of the COUGH group (*n* = 7) limited our analysis and the interpretation of non-significant differences between the groups. The small sample size also limited our ability to provide reliable individual parameter estimates for the IOS measurements in the multiple regression analysis predicting DI-index. It is possible that further differences exist, and a larger sample would help verify our observations. The age and demographics of the recruited CA, CVA, and COUGH participants are typical of the patient population referred to tertiary cough/asthma clinics. Furthermore, the CONTROL group was recruited to match the other three groups in terms of age and sex, which may limit the generalizability of our findings. For most measurements, we presented the % predicted values to account for the age- and sex-related differences, but it is possible that further differences exist.

Our CA group consisted of participants with mostly mild-to-moderate airway hypersensitivity to methacholine (7/11), whereas all CVA participants had borderline-to-mild airway hypersensitivity. It is possible that the inclusion of participants with more severe asthma may yield different results. There was also some overlap in asthma severity (in terms of PC_20_) between the CA and CVA groups. Even within the COUGH and CONTROL groups, there was a variable response to methacholine (Sood et al., 2017), which could affect the interpretation of our results. Interestingly, cough with normal airway sensitivity has also been described during mannitol challenge (Koskela et al., 2004, 2018; Turcotte and Lougheed, 2011) and hypertonic saline and hypertonic histamine challenges (Koskela et al., 2005). Future studies could use another stimulus (e.g., mannitol and cold air) to elucidate further pathophysiologic differences between the four groups.

Some participants in the CA, CVA, and COUGH groups were using inhaled corticosteroids as maintenance therapy and did not withhold these prior to methacholine challenge. Inhaled corticosteroid use, even at low doses, is known to suppress airway inflammation in asthma and, therefore, may be associated with a decreased sensitivity to methacholine (Fujimura et al., 2005). We used a methacholine threshold of 16 mg/mL to minimize any resulting diagnostic misclassification based on previous work by Luks et al. (2010). However, approximately 73% of participants with CA, 40% with CVA, and 43% with COUGH were taking an inhaled corticosteroid prior to study enrollment, which could have attenuated their responses and/or sensitivity to methacholine. Medication use may also have impacted our participants’ response to DIs as demonstrated by Bel et al. (1990), where prior use of anti-inflammatory (leukotriene inhibitor) therapy enhanced of DI-induced bronchodilation during methacholine challenge.

## Conclusion

Our main finding is that the bronchodilating effect of DIs is impaired in COUGH, compared to healthy normals and asthma. Our IOS data have provided novel insights into the involvement of peripheral airways in the bronchodilating effects of DIs. Decreased Fres and increased R5–R20 are predictive of a decreased ability to bronchodilate after a DI. In COUGH, dyspnea intensity and cough frequency in response to high-dose methacholine are comparable to CA and CVA, respectively, despite mechanical responses comparable to healthy normals. COUGH is a distinct and clinically relevant airway disorder related to impaired peripheral airway function.

## Data Availability Statement

The data that support the findings of this study are available on request from the corresponding author NS, 0ns10@queensu.ca. The data for the control participants are available on request at doi: 10.5683/SP2/HECUFP.

## Ethics Statement

The studies involving human participants were reviewed and approved by the Queen’s University Health Sciences and Affiliated Teaching Hospitals Research Ethics Board (HSREB). The patients/participants provided their written informed consent to participate in this study.

## Author Contributions

NS performed experiments, analyzed the data, interpreted results of experiments, prepared figures and tables, drafted the manuscript, edited and revised the manuscript, and approved the final version of the manuscript. NW performed experiments, analyzed the data, and approved the final version of the manuscript. AD analyzed the data, interpreted results of experiments, prepared figures and tables, and approved the final version of the manuscript. TW and TF performed experiments and approved the final version of the manuscript. JF edited and revised the manuscript, and approved the final version of the manuscript. ML conceived and designed research, interpreted results of experiments, drafted, edited, and revised the manuscript, and approved the final version of the manuscript. All authors contributed to the article and approved the submitted version.

## Conflict of Interest

ML has received grants outside the submitted work paid directly to Queen’s University from the Ontario Lung Association/Ontario Thoracic Society, the Government of Ontario’s Innovation Fund, AllerGen NCE, Queen’s University, AstraZeneca, GlaxoSmithKline, Hoffman-La Roche, Janssen, and Novartis, honoraria from the Ontario Lung Association for preparation and review of educational materials, an honorarium from the Canadian Thoracic Society for co-development and co-presentation of the Severe Asthma PREP course, and honoraria from AstraZeneca for participation in the Precision Program Advisory Board. The remaining authors declare that the research was conducted in the absence of any commercial or financial relationships that could be construed as a potential conflict of interest.
