# Supplementary material for: Methacholine-Induced Cough in the Absence of Asthma: Insights From Impulse Oscillometry
Source: Front Physiol. 2020 Oct 6;11:554679. doi: 10.3389/fphys.2020.554679 (PMC7573225; doi:10.3389/fphys.2020.554679)
Supplement: Supplementary file 1 [file Data_Sheet_1.pdf]

Supplementary Material

1 Supplementary Methods: Assessing IOS Measures as Predictors of DI Index

Before modelling, the IOS parameters were transformed using natural logarithms to achieve a more Gaussian distribution. Since X5 values are negative, the negative sign was removed prior to log transformation, and the resulting log-transformed values were negated for analysis.

Due to the small sample size, we used principal components (PCs) to reduce the 22 covariates (age, sex, height, BMI, spirometry measures (FEV_1, ΔFEV_1, $\%pr\Delta FEV_1$, FVC, ΔFVC, %prΔFVC, FEV_1/FVC, PEF, $FEF_{50\%}$, $FEF_{25\text{-}75\%}$, $FEF_{75\%}$) and lung volume measures (TLC, RV, RV/TLC, FRC, ERV, IC, IC/TLC)) to five orthogonal PCs with an eigenvalue $\geq$1. These five PCs were then used as covariates in an attempt to approximately control for all 22 underlying variables.

We first examined the raw bivariate associations between the IOS measures and DI-Index using the Pearson correlation coefficient, and then re-examined the correlation after controlling for the five PCs using Pearson's partial correlation. In addition to the raw p-values, we reported false discovery rates to account for the six IOS measures tested.

We used the adjusted R^2 and F-test from multiple linear regression to assess the significance of the association between the six IOS variables collectively and the DI index before and after controlling for the five PCs representing the 22 covariates. Even though the ratio of sample size to predictors was low, the adjusted R^2 and F-test remain valid as long as the model assumptions are met since no model selection has taken place. However, due to the small sample size and high correlation between the IOS variables, the individual model parameters are not reliable.

As an exploratory analysis to attempt to identify which specific IOS variables may be independently predictive of DI index, we ran a cross-validated model selection forcing the 5 PCs in the models and selecting among the six IOS variables using stepwise selection with entry and exit criteria of p=0.15 with selection truncated if the leave-out-one cross-validation resulted in an increasing predicted residual sum of squares. To assess the stability of these results, we performed this cross-validated truncated stepwise selection in 10,000 bootstrap samples and reported the frequency of selection and parameter inter-quartile range for the six IOS variables.

2 Supplementary Figure

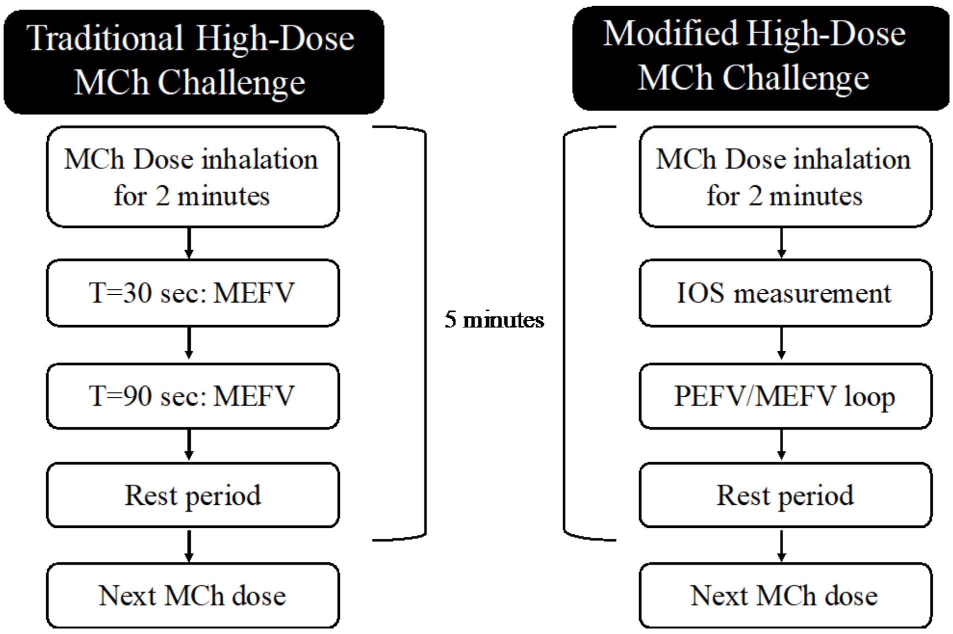

Figure S1. Difference between the traditional and modified high-dose methacholine protocol design. IOS = impulse oscillometry; MCh=methacholine; MEFV = maximal expiratory flow volume curve; PEFV = partial expiratory flow volume curve.

s

3 Supplementary Tables

Table S1. Pearson correlations between 22 variables used to create five principal components (PCs) for adjustment.

	Gender	Age (years)	Height (cm)	BMI (kg/m^2)	FEV$_1$ (L)	ΔFEV$_1$ (L)	ΔFEV$_1$ (%pr)	FVC (L)	ΔFVC (L)	ΔFVC (%pr)	FEV$_1$/ FVC	PEF (L/s)	FEF$_{50\%}$ (L/s)	FEF$_{25-75\%}$ (L/s)	FEF$_{75\%}$ (L/s)	TLC (L)	RV (L)	RV/ /TLC	FRC (L)	ERV (L)	IC (L)	IC/ TLC
**Gender**	1.00	-0.20	0.50	-0.03	0.54	-0.09	0.23	0.55	0.05	0.29	0.04	0.51	0.32	0.42	0.42	0.58	0.10	-0.28	0.26	0.47	0.51	0.15
**Age (years)**	-0.20	1.00	-0.35	0.01	-0.47	-0.12	-0.35	-0.44	-0.24	-0.36	-0.22	-0.49	-0.41	-0.45	-0.51	-0.24	0.17	0.39	-0.14	-0.24	-0.28	-0.22
**Height (cm)**	0.50	-0.35	1.00	-0.31	0.63	-0.20	0.23	0.71	-0.01	0.30	-0.02	0.52	0.40	0.44	0.48	0.69	-0.08	-0.56	0.43	0.59	0.56	0.08
**BMI (kg/m^2)**	-0.03	0.01	-0.31	1.00	-0.30	-0.19	-0.33	-0.33	-0.31	-0.41	-0.04	-0.27	-0.22	-0.24	-0.18	-0.10	0.27	0.35	-0.16	-0.38	-0.02	0.11
**FEV$_1$ (L)**	0.54	-0.47	0.63	-0.30	1.00	0.35	0.75	0.93	0.54	0.77	0.37	0.89	0.78	0.90	0.83	0.66	-0.17	-0.57	0.31	0.64	0.73	0.37
**ΔFEV$_1$ (L)**	-0.09	-0.12	-0.20	-0.19	0.35	1.00	0.84	0.15	0.86	0.71	0.58	0.33	0.45	0.52	0.44	-0.23	-0.16	0.00	-0.10	-0.09	-0.06	0.26
**ΔFEV$_1$ (%pr)**	0.23	-0.35	0.23	-0.33	0.75	0.84	1.00	0.57	0.84	0.92	0.62	0.68	0.69	0.82	0.72	0.20	-0.17	-0.30	0.04	0.25	0.37	0.41
**FVC**	0.55	-0.44	0.71	-0.33	0.93	0.15	0.57	1.00	0.44	0.69	0.01	0.81	0.59	0.69	0.66	0.80	-0.08	-0.58	0.52	0.79	0.71	0.21
**ΔFVC**	0.05	-0.24	-0.01	-0.31	0.54	0.86	0.84	0.44	1.00	0.90	0.39	0.51	0.49	0.59	0.55	-0.03	-0.19	-0.14	0.13	0.23	0.07	0.24
**ΔFVC (%pr)**	0.29	-0.36	0.30	-0.41	0.77	0.71	0.92	0.69	0.90	1.00	0.37	0.71	0.59	0.76	0.70	0.30	-0.12	-0.32	0.22	0.43	0.38	0.33
**FEV$_1$/ FVC**	0.04	-0.22	-0.02	-0.04	0.37	0.58	0.62	0.01	0.39	0.37	1.00	0.36	0.68	0.69	0.59	-0.23	-0.25	-0.10	-0.44	-0.24	0.17	0.46
**PEF (L/s)**	0.51	-0.49	0.52	-0.27	0.89	0.33	0.68	0.81	0.51	0.71	0.36	1.00	0.75	0.82	0.76	0.60	-0.04	-0.44	0.34	0.56	0.61	0.27
**FEF$_{50\%}$ (L/s)**	0.32	-0.41	0.40	-0.22	0.78	0.45	0.69	0.59	0.49	0.59	0.68	0.75	1.00	0.88	0.79	0.30	-0.31	-0.50	0.03	0.37	0.50	0.37
**FEF$_{25-75\%}$ (L/s)**	0.42	-0.45	0.44	-0.24	0.90	0.52	0.82	0.69	0.59	0.76	0.69	0.82	0.88	1.00	0.93	0.39	-0.21	-0.44	0.06	0.37	0.62	0.48
**FEF$_{75\%}$ (L/s)**	0.42	-0.51	0.48	-0.18	0.83	0.44	0.72	0.66	0.55	0.70	0.59	0.76	0.79	0.93	1.00	0.37	-0.25	-0.48	0.11	0.44	0.55	0.39

**TLC (L)**	0.58	-0.24	0.69	-0.10	0.66	-0.23	0.20	0.80	-0.03	0.30	-0.23	0.60	0.30	0.39	0.37	1.00	0.34	-0.31	0.61	0.65	0.73	0.01
**RV (L)**	0.10	0.17	-0.08	0.27	-0.17	-0.16	-0.17	-0.08	-0.19	-0.12	-0.25	-0.04	-0.31	-0.21	-0.25	0.34	1.00	0.76	0.37	-0.14	0.04	-0.23
**RV/TLC**	-0.28	0.39	-0.56	0.35	-0.57	0.00	-0.30	-0.58	-0.14	-0.32	-0.10	-0.44	-0.50	-0.44	-0.48	-0.31	0.76	1.00	-0.06	-0.56	-0.40	-0.20
**FRC (L)**	0.26	-0.14	0.43	-0.16	0.31	-0.10	0.04	0.52	0.13	0.22	-0.44	0.34	0.03	0.06	0.11	0.61	0.37	-0.06	1.00	0.65	0.01	-0.57
**ERV (L)**	0.47	-0.24	0.59	-0.38	0.64	-0.09	0.25	0.79	0.23	0.43	-0.24	0.56	0.37	0.37	0.44	0.65	-0.14	-0.56	0.65	1.00	0.35	-0.18
**IC (L)**	0.51	-0.28	0.56	-0.02	0.73	-0.06	0.37	0.71	0.07	0.38	0.17	0.61	0.50	0.62	0.55	0.73	0.04	-0.40	0.01	0.35	1.00	0.64
**IC/TLC**	0.15	-0.22	0.08	0.11	0.37	0.26	0.41	0.21	0.24	0.33	0.46	0.27	0.37	0.48	0.39	0.01	-0.23	-0.20	-0.57	-0.18	0.64	1.00

PEF = peak expiratory flow; $FEF_{50\%}$ = forced expiratory flow at 50% of the forced vital capacity; $FEF_{75\%}$ = forced expiratory flow at 75% of the forced vital capacity; FRC = functional residual capacity; ERV = expiratory reserve volume. For the remaining definitions of abbreviations, see Table 1.

Table S2. Principal component (PC) analysis.

Number of Extracted PCs	Eigenvalue	Variance explained of 22 covariates	Variance explained of DI-index
1	9.8	45%	13%
2	4.0	63%	14%
3	2.2	73%	14%
4	1.9	81%	23%
5	1.0	85%	32%
6	0.8	89%	33%
7	0.6	92%	34%
8	0.5	94%	36%
9	0.3	96%	37%
10	0.2	97%	43%
11	0.2	98%	43%
12	0.1	98%	44%
13	0.1	99%	48%
14	0.1	99%	49%
15	0.1	99%	56%
16	0.1	100%	56%
17	0.0	100%	59%
18	0.0	100%	59%
19	0.0	100%	60%
20	0.0	100%	61%
21	0.0	100%	63%
22	0.0	100%	63%

Table S3. Regression model evaluating only IOS measures as predictors for DI Index (n=42).

	Degrees of freedom		Mean Square		Model Test		Variance explained	
**Model Description**	**Model**	**Error**	**Model**	**Error**	**F-Value**	**p-value**	**R^2**	**adjR^2**
6 IOS variables	6	34	0.68	0.25	2.77	0.027	33%	21%
5 PCs	5	34	0.63	0.19	3.24	0.017	32%	22%
6 IOS variables + 5 PCs	11	27	0.51	0.15	3.46	0.004	58%	42%
Test for addition of 6 IOS variables to model with 5 PCs	6	27	0.43	0.15	2.93	0.025	26%	20%

IOS = impulse oscillometry; PC = principal component; adj = adjusted.

Table S4. Summary of stepwise selection from 10,000 bootstrap samples.

Parameter	Included in model (%)	Mean Estimate	Standard Deviation	Estimate Quantiles		
				25%	Median	75%
Intercept	100	-4.37	2.43	-6.14	-4.65	-2.56
**Forced into model**						
PC1*	100	0.10	0.03	0.08	0.10	0.12
PC2*	100	0.02	0.03	0.00	0.02	0.04
PC3*	100	-0.03	0.05	-0.06	-0.03	0.00
PC4*	100	-0.06	0.06	-0.09	-0.06	-0.03
PC5*	100	-0.03	0.08	-0.08	-0.02	0.03
**Impulse Oscillometry**						
Log(R5)	17	-0.17	0.59	0.00	0.00	0.00
Log(R20)	27	0.24	0.51	0.00	0.00	0.34
Log(R5-R20)	47	-0.25	0.34	-0.52	0.00	0.00
Log(AX)	19	-0.09	0.32	0.00	0.00	0.00
-Log(-X5)	16	0.01	0.25	0.00	0.00	0.00
**Log(Fres)**	**90**	**1.70**	**0.95**	**0.95**	**1.80**	**2.34**

Bolded variables were selected in ≥50% of 10,000 bootstrap samples.
*forced into the model
PC = principal component; R5 = resistance at 5 Hz (total respiratory resistance); R5-R20 = frequency dependence of Rrs (R5 minus R20, peripheral respiratory resistance); R20 = resistance at 20 Hz (central respiratory resistance); X5 = reactance at 5 Hz (peripheral reactance); AX = area under the reactance curve below resonant frequency; Fres = resonant frequency.
